# A Prognosis Classifier for Breast Cancer Based on Conserved Gene Regulation between Mammary Gland Development and Tumorigenesis: A Multiscale Statistical Model

**DOI:** 10.1371/journal.pone.0060131

**Published:** 2013-04-02

**Authors:** Yingpu Tian, Baozhen Chen, Pengfei Guan, Yujia Kang, Zhongxian Lu

**Affiliations:** Xiamen City Key Lab of Metabolism Disease & Metabolic Disease Research Center, Institute for Biomedical Research, Xiamen University, Xiamen, Fujian, China; Institute of Molecular and Cell Biology, Singapore

## Abstract

Identification of novel cancer genes for molecular therapy and diagnosis is a current focus of breast cancer research. Although a few small gene sets were identified as prognosis classifiers, more powerful models are still needed for the definition of effective gene sets for the diagnosis and treatment guidance in breast cancer. In the present study, we have developed a novel statistical approach for systematic analysis of intrinsic correlations of gene expression between development and tumorigenesis in mammary gland. Based on this analysis, we constructed a predictive model for prognosis in breast cancer that may be useful for therapy decisions. We first defined developmentally associated genes from a mouse mammary gland epithelial gene expression database. Then, we found that the cancer modulated genes were enriched in this developmentally associated genes list. Furthermore, the developmentally associated genes had a specific expression profile, which associated with the molecular characteristics and histological grade of the tumor. These result suggested that the processes of mammary gland development and tumorigenesis share gene regulatory mechanisms. Then, the list of regulatory genes both on the developmental and tumorigenesis process was defined an 835-member prognosis classifier, which showed an exciting ability to predict clinical outcome of three groups of breast cancer patients (the predictive accuracy 64∼72%) with a robust prognosis prediction (hazard ratio 3.3∼3.8, higher than that of other clinical risk factors (around 2.0–2.8)). In conclusion, our results identified the conserved molecular mechanisms between mammary gland development and neoplasia, and provided a unique potential model for mining unknown cancer genes and predicting the clinical status of breast tumors. These findings also suggested that developmental roles of genes may be important criteria for selecting genes for prognosis prediction in breast cancer.

## Introduction

Human breast tumorigenesis is thought to require multiple gene mutations, and different tumors often have distinct molecular abnormalities [1]. So, classification of tumor type and stage can now be assessed by the gene expression patterns during the tumor developmental process [2], [3]. Thus, the improved methods for early diagnosis, molecularly targeted therapy, and prognosis assessment have been emphasizes on the identification of novel genes or small gene subsets [4]–[6], and a small number of prognostic prediction gene sets, such as MammaPrint [7]–[10], Mapquant [5], [11], [12], and Theros [13]–[15], have been identified. Especially, MammaPrint (a 70 genes subset) showed strong prognosis value and is the first and only prognosis gene set for breast cancer that have been approved by the FDA [4]. However, these small prognosis gene sets were identified from the gene expression profiles of different breast cancer samples, and they have almost no common genes [4]. As a result, their prognosis value may only apply to special types of tumors and are not yet adequate to ensure significant improvement in the clinical outcome of breast cancer [3], [4], [16], [17]. Thus, new powerful screening models are still required for effective diagnosis and treatment of breast cancer.

Pathways relevant to cancer were linked to those found in normal organ development over one hundred years ago [18], [19]. The processes of development and neoplasia both involve cellular alterations such as proliferation, differentiation, migration and invasion, neovascularization and apoptosis [1], [20]. Therefore, it is not surprising that accumulating evidence now suggests that normal development and cancer share molecular properties [21], [22]. Two recent studies used statistically-based computational approaches to explore the relationships between gene expression in mouse cerebellum and intestine with similar expression in respective human cancers (i.e., medulloblastoma and colon cancer) [23], [24]. In general, genes that were up-regulated in cancerous tissues also tended to be those that were active in the “growth” stages of the corresponding normal developmental process and that had a significantly high probability of mediating early organ development. In contrast, genes that were down-regulated in tumors tended to correspond to genes found expressed at later stages of development in normal tissues. Both of these patterns indicated a recapitulation of tissue-specific developmental programs in the cancerous tissue, suggesting that these types of bioinformatics approaches may be useful for identifying candidate biomarkers and therapeutic targets [23], [24].

The mammary gland is a unique mammalian developmental system, and starts its cyclical developmental phase with pregnancy when the gland undergoes dramatic changes in size, shape, and function with the continuous production and differentiation of epithelial cells, followed by apoptosis of epithelial cells upon weaning [25], [26]. These frequent cycles of proliferation and apoptosis require an array of molecular effectors that depend on tight regulatory mechanisms [25], [27]. Perturbations in any of the pathways involved in these types of regulatory networks may make the mammary gland susceptible to neoplasitc transformation [1], [20]. As a result, the risk of sporadic breast cancer is thought to be heavily influenced by developmental factors and to be modified by lifestyle and the environment [1], [20]. Genes involved in normal development are also frequently involved in breast tumorigenesis [28]–[30]. However, these evidences has usually been gathered in piecemeal fashion [28]–[30]. No systematic analysis has been performed to explore the global characteristics of the overlap in gene expression between the developing tissues and cancer in the mammary gland. We also don’t know whether the genes associated with mammary gland development can be a resource to identify cancer associated genes and to refine the prognosis genes sets.

In the present study, we analyzed the intrinsic correlation between the gene expression profiles of mammary gland development and breast cancer using a multiscale statistical approach, thus defining a subset of common genes expressed both during mammary gland development and in breast tumors. Then, we assessed the value of genes associated with development in the prediction of clinical outcome of human breast cancer. Our study is the first global analysis to demonstrated common molecular mechanism between mammary gland development and tumorigenesis. We provide strong evidence that the expression profiles of developmental genes can be used in a model for selecting genes to predict prognosis in breast cancer.

## Results

### Definition of a Mammary Gland Developmentally Associated Gene Subset

**Figure 1:**
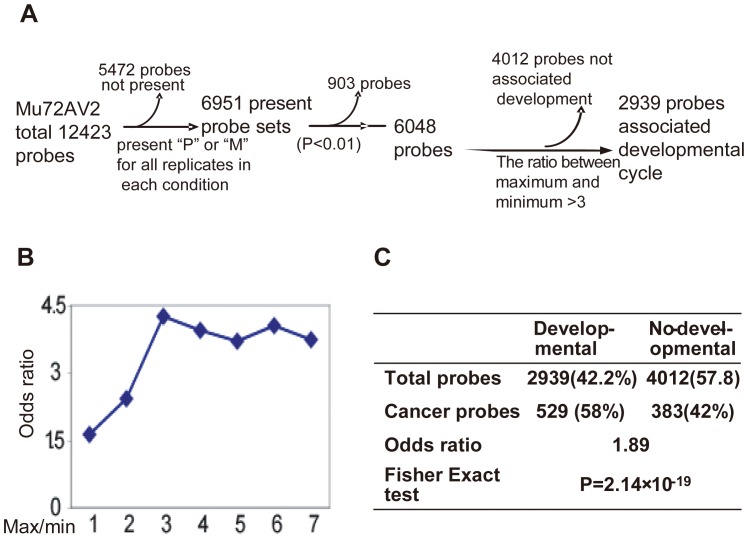
Definition of mammary gland developmentally associated genes. Overview of data processing for defining the developmentally associated gene subset was discribled in **A.** The probes were filtered systematically with different cutoffs:p value of gene expression among different time points and the optimal fold of maximum/minimum expression of a gene at different developmental time points, which should have a maximum Odds ratio of literature-based mammary gland-cycle associated genes in developmentally associated and non-developmentally associated genes subset. A higher Odds ratio means that a greater number of developmental genes were correctly classified. The figure **B** shows the curve of Odd ratios in the developmentally associated and non-developmentally associated genes subset defined by a cutoff with different ratio of maximum to minimum expression of each gene at different time points in the developmental progress. **C.** Fisher exact test to assess the frequency of validated cancer gene expression in the group of mammary gland developmentally associated genes. The validated cancer genes were obtained from previously published papers (File S5).

Firstly, a “Present” genes dataset containing 6951 probe sets was extracted from a published mammary gland gene profiling database after non-expressed genes and genes expressed at a low level (“Absent” probes) were filtered out (See **Materials and Methods**).(Fig. 1A) [31], Then, we calculated the p value (ANOVA assay) of gene expression among different time points and used as a first cutoff the significant changes of gene expression during the developmental cycle. However, we found that this cutoff is likely too unselective because probes with a *p* value less than 0.01 was 6048 and covered more than 90% of the presented genes.

So, we then employed the ratio of maximum expression to minimum expression of a gene at different developmental time points as a second cutoff to increase specificity and narrow down the list of mammary gland developmentally associated genes. Here, we compiled a list of literature-based mammary gland-cycle associated genes (File S1) to assess the quality of defined developmentally associated gene list. To quantify the quality of the developmentally associated genes dataset, we employed the odds ratio of literature-based mammary gland-cycle associated genes in the developmentally and non-developmentally associated genes subset. A higher odd ratio means that developmentally associated genes subset contain more developmentally associated genes, with few non-developmental associated genes.

With increasing of the ratio of maximum expression to minimum expression of a gene, we found that the odds ratio of literature-based mammary gland-cycle associated genes reaches a peak of 4.27 at 3 fold of max/min expression (Fig. 1B). Thus, genes with 3 fold of max/min expression was defined as mammary gland “developmentally associated gene”, which contained 2939 probes that varied statistically significantly across our time course (*P*<0.01) and had more than a 3 fold of max/min expression (Fig. 1A, File S2).

The developmentally associated gene dataset is robust because, although it only covers 42.2% of expressed probes, it contains more than 75% of the genes in the literature-based mammary gland cycle associated genes (File S1) (the highest Odds ratio: 4.27, *P* value = 4.4*10^−11^) (Fig. 1B). Most of the biological processes and molecular pathways involved in mammary gland development were enriched in this developmentally associated genes list (File S3). The most highly enriched biological processes and pathways were fatty acid and lipid metabolism, cell cycle, and cell adhesion, which corresponded to mammary gland milk production, epithelial growth, and invasion, respectively (File S3). Cell differentiation, angiogenesis, and blood vessel development were also enriched in the developmentally associated gene list. Using a literature-based cancer modulated gene list (File S4), we found that the developmentally associated genes list also contained more cancer-associated genes than the non-developmentally associated genes. The Odds ratio for literature-based cancer modulated genes is 1.89 (covers 58% of cancer genes, *P* value = 2.14*10^−19^) (Fig. 1C). The enrichment of genes mediating tumorigenesis among developmentally associated genes supports the hypothesis that there is a correlation between gene regulation occurring during development and in tumorigenesis, most likely based on common cellular processes such as proliferation, invasion, migration, differentiation, and apoptosis.

**Figure 2:**
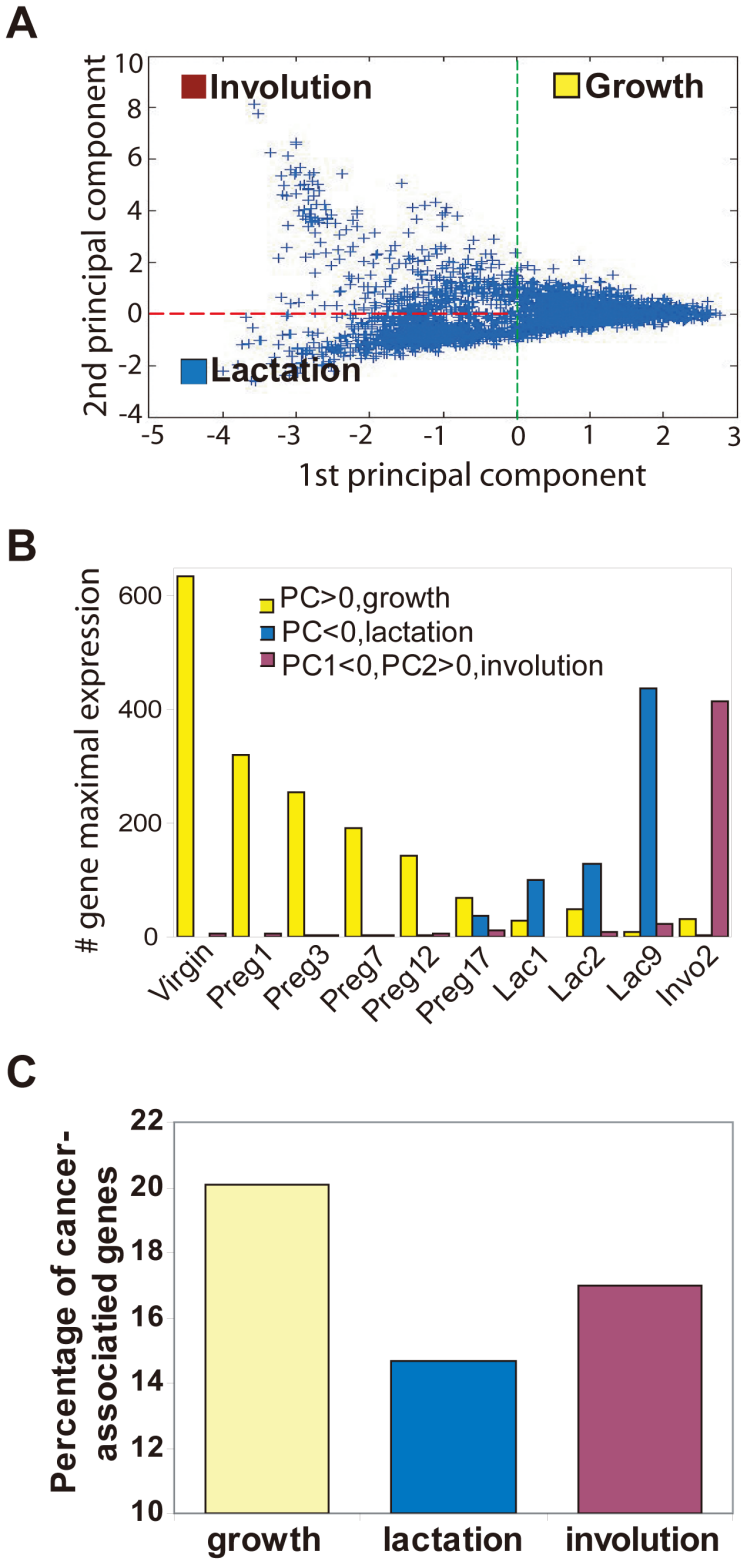
Identification of genes associated with the developmental phases of growth, lactation, and involution among the mammary gland developmentally associated gene subset. **A.** The developmentally associated genes were clustered into three groups by Principal Component Analysis. Expression profiles of genes in mammary pregnancy cycle are represented as dots in PC1 (1^st^ principal component axis) and PC2 (2^nd^ principal component axis). All probe sets were grouped into three groups: growth (PC1>0), involution (PC1<0&PC2>0) and lactation (PC1<0&PC2<0) based on the number of genes that have peak expression at a particular developmental time (showed in B). **B.** The time of peak expression for each developmentally associated gene was plotted on a histogram and classified according to the developmental phase (growth, yellow; lactation, blue; involution, purple). The column represents the number of genes that have peak expression at a particular developmental time. **C.** The frequency of a literature-based cancer modulated genes in the gene subsets associated with the three different stages of mammary gland development. The “growth” group contained more literature-based cancer modulated genes (20%) than the “lactation” (14.7%) and the involution (17%) groups (*p*<0.05).

To character the developmentally associated gene dataset, all probe sets were grouped into three groups based on their PC1 and PC2 value with principal component analysis (PCA) (File S5) (Fig. 2A). Then, we assess the expression pattern of the probe in each group, and found that their expression were correlation to the stage of mammary gland development. Most of genes (PC1>0) have a peak expression in mammary gland during the stage of virgin and early pregnancy when mammary gland epithelial cells undergo active proliferation. So, the gene group with positive PC1 was named the “growth” genes (Fig. 2B, yellow columns). In a similar way, the genes group (PC1<0 and PC2<0) was regarded as the “lactation” gene because their expression exhibit a peak during the lactation phases (Fig. 2B, blue column), while the “involution” genes (PC1<0 and PC2>0) represent the genes have a peak expression during the involution stage (Fig. 2B, purple column). This classification was also supported by gene ontology analysis (File S3). In the growth group, the most highly enriched processes were cell adhesion, cell cycle, and response to external stimuli; all processes that are active in the growth phase of mammary gland development. In the lactation group, the most highly enriched processes and pathways were metabolism, biosynthesis, and cell differentiation, consistent with the terminal differentiation and biological function of mammary epithelial cells. In the involution group, the most enriched process was homeostasis.

We also assessed the neoplasitc character of the genes in these three groups. In the “growth” group, the percentage of literature-based cancer modulated genes (File S4) was 20%, which was significantly higher than the percentage in the “lactation” (14.7%) and the involution (17%) groups (Fig. 2C) (*p*<0.05). These findings supported the idea that gene batteries expressed during different mammary gland developmental phases may carry out distinct biological processes, some of which may also be important for oncogenesis. During the early phase of mammary gland development, gene expression leads to cell growth, invasion, and neovascularization–all processes that strongly support tumor formation and growth. Genes expressed in the involution stage are involved in apoptosis, which should be suppressed during tumorigenesis. Therefore, it was not surprising that cancer genes were also enriched during this phase. In contrast, cancer genes were underrepresented among the differentiation genes important for lactation.

### The Expression Profiles of Mammary Gland Developmental Genes can Correctly Classify the Different Characters of Breast Tumors

**Figure 3.The:**
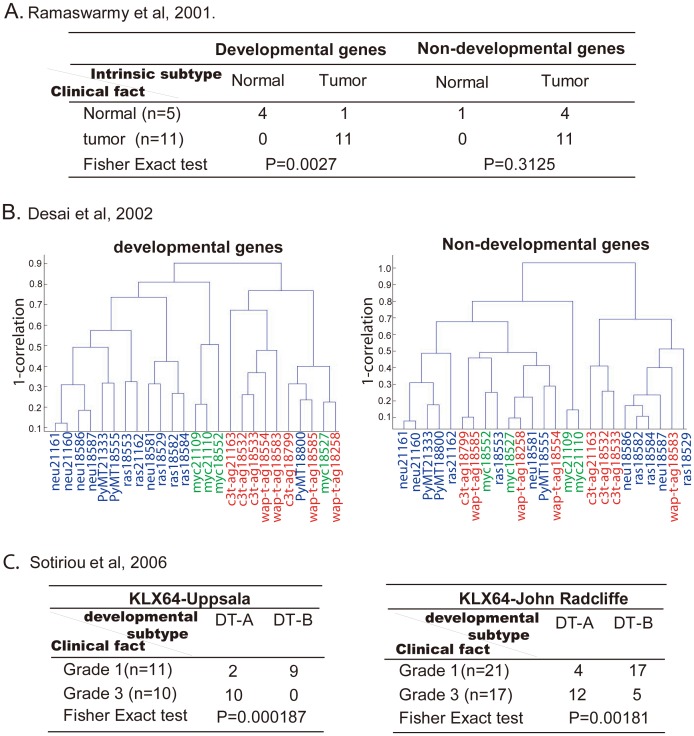
expression profiles of mammary gland developmental genes in breast tumors reflect the characteristics of the tumors. Tumors samples include 5 normal tissues and 11 breast cancer samples (**A**), 25 mouse mammary gland tumors from six human oncogenic transgenetic mice (**B**), as well as two groups of grade 1 and grade 3 human breast tumors (**C**). Tumors were first classified based on the expression profiling of mammary gland developmentally associated genes by unsupervised classification with hierarchical clustering algorithm. Then, accuracy of classification was assessed with Fisher exact test. The non-developmental gene subset was included as a control.

Previous studies have shown that tumors at different stages of development, such as precancerous, invasive and metastatic, exhibit unique expression profiles [8], [11], [32], [33]. Based on our finding that developmentally associated mammary gland genes are enriched in breast cancer, we hypothesized that mammary gland developmental associated genes might have differential representation in tumors of different stages. To test this idea, we carried out an unsupervised classification, using a hierarchical clustering algorithm, in three breast cancer microarray databases (Fig. 3).

First, we used a gene expression profiling datasets of 5 normal breast and 11 breast cancers to investigate whether breast cancer can be distinguished from normal tissue by the expression pattern of mammary gland developmentally associated genes [34]. All 11 tumor samples (100%) and 4 normal tissues (80%) were correctly classified (*p*<0.01) (Fig. 3A). In contrast, non-developmental genes do not distinguish tumor from normal breast tissue (*P*>0.05, Fig. 3A), supporting the specificity of the findings.

Second, we tested the possibility that expression of the developmentally associated genes could correctly classify tumors originating through distinct oncogenic mechanisms. Desai et. al. [35] profiled gene expression in breast tumors from six transgenic mice and defined an “altered” cancer gene subset by F test. They then used the average linked hierarchical clustering of the altered genes to demonstrate that the tumor types separated into three groups: 1) C3(1)/simian virus 40(SV40) T/t antigen (C3-t-ag) and WAP-SV40 T/t antigen (WAP-t-ag) tumors, in which both checkpoints of the cell cycle are defective by inactivation of p53 and pRb; 2) MMTV-c-myc tumors, in which cell cycle mediated genes, transcription factors, and ribosomal RNA genes were induced; 3) MMTV-neu, MMTV-Ha-Ras, and MMTV-polyoma middle t antigen (PyMT) tumors, in which the expression of certain cell cycle genes (cyclin D1,cdk-2 and E2F) were induced, but not genes involved in G2/M transition or those affecting DNA replication. Interestingly, developmentally associated genes also classified tumors into three similar groups:SV40 T/t antigen (red), c-myc (green), and Neu-ras-PyMT (blue) (Fig. 3B, left panel). Only a single MMTV-c-myc and MMTV PyMT induced tumor was misclassified as a SV40 T/t antigen tumor. In contrast, the expression profiling of non-developmental genes failed in correctly classifying the tumors (Fig. 3B, right panel).

Third, we tested whether tumor progression status can be correctly determined based on the expression of developmentally associated genes. Sotiriou et al [11] analyzed the gene expression profiles for breast cancer of three different grades and found that histological grades 1 and 3 were associated with distinct gene expression patterns. A grade associated gene subset that was refined from the training set (KJX64) also accurately predicted the histological grade of breast tumors in several validation sets (KJ125, NCI, STNO and NK12) [11]. We assessed whether the developmentally associated genes could also predict histological grade of breast cancer as was shown for the “grade associated gene” in Sotiriou et al [11]. Expression profiles of developmentally associated genes were quite successful in classifying the histological status of breast tumors in the KJX64-Uppsala and KJX64-John Radcliffe data sets (Fig. 3C). In the KJX64-Uppsala set, 9 tumors of grade 1 and 10 tumors of grade 3 were correctly predicted (19, 90.48% of total 21 patients) (Fisher exact test *P*<0.001). In the KJX64-John Radcliffe tumors set, 29 or 76.3% of total 38 tumors were correctly predicted.

In conclusion, the expression profiles of developmentally associated genes in breast tumor tissues conferred a unique ability to classify different tumor characters.

### Derivation of a Prognosis Classifier from the Regulated Genes Common to Both Breast Development and Tumorigenesis

One of main goals of gene expression profiling in breast cancer is to use the information to predict prognosis. Based on the results described above, we hypothesized that mammary gland developmentally associated genes may be useful for prognosis prediction, leading us to develop a “prognosis classifier” from this set of genes.

**Figure 4:**
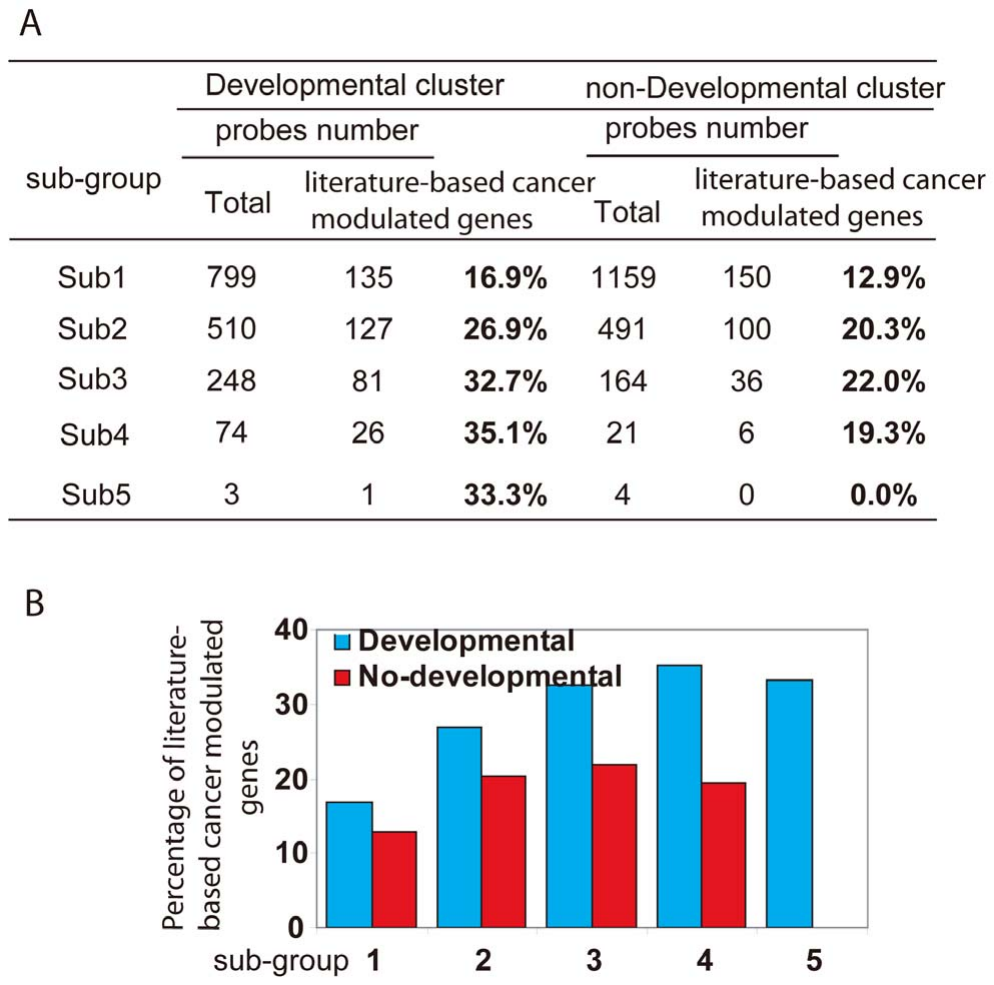
Defining the 835 prognosis classifier from the developmentally associated genes based on their expression in tumors. For each developmentally associated gene, we first counted the number of the breast cancer datasets in which it was “altered” in expression. Based on this database number, all developmental genes were then grouped into six subsets (Sub0, Sub1, Sub2, Sub3, Sub4, and Sub5). The percentage of a literature-based cancer modulated genes in each subset is shown in table (**A**) and histogram (**B**). The results of non-developmental genes with same assay method are shown as a control. The details are described in the text.

We first optimized the developmentally associated genes subset based on the frequency of altered gene expression in five breast cancer gene expression databases. Each developmentally associated genes was grouped into six subsets (Sub0, Sub1, Sub2, Sub3, Sub4, and Sub5) based on the number of breast cancer datasets in which the gene expression was significantly altered. The percentage of literature-based cancer modulated genes was then assessed in each subset (Fig. 4). As expected, the enrichment of cancer genes in the subsets increased in proportion to the number of breast cancer databases with altered expression (Fig. 4). The subsets of genes in which expression was altered in two cancer databases (Sub2) contained 26.9% of the cancer-associated genes, which was significantly higher than that of Sub0 or Sub1. The Sub3, Sub4, and Sub5 also contained 32.7, 35.1, and 33.3% of the cancer-associated genes, respectively.

We selected 835 mammary gland developmentally associated genes as a prognosis prediction subset based on altered expression in at least two primary breast cancer databases (referred to as the “835 prognosis classifier”). The 835 prognosis classifier contained 28.4% of the literature-based mammary gland cycle associated genes (File S3) and 44.5% of a literature-based cancer modulated genes (File S4). Gene ontology analysis showed the 835 prognosis classifier decreased the representation of metabolic genes (fatty acid, lipid, carboxylic acid), which appear to be related to milk production in the mammary gland and are less likely to be involved in tumor formation. In contrast, cell cycle, cell proliferation, cell adhesion, cell growth and death, angiogenesis, and blood vessel development genes were enriched–all processes that are shared between the developmental and tumorigenesis processes (File S7). The 835 prognosis classifier contained many cancer genes, such as Trp53, Myc, Kit, RB1, EGFR, CCND1, and CCND2 [36], and several genes involved in growth factor signaling, including TGFβ (TGFBI, TGFb1, TGFB2, TGFB3), IGF (IGF1, IGF2, IGFBP6), PDGF (PDGFB, PDGFRA), EGF (EGFR) and VEGF (VEGFc, VEGFA), all of which are involved in both mammary gland development and carcinogenesis (Table 1).

**Table 1:** The enrichment of ontology in 835 intrinsic genes.

Process and pathway	Genes
Cell proliferation/cell cycle (149 )	VEGFC, Vegfa, TTK, Trp53#, TOP2A, TNFRSF1B, THRA, TGFBI, TGFB3, TGFB2, TGFB1, TACC3, STAT1, SSR1, SPP1, Snk, SET, SESN1, S100A11, RRM2, RRM1, RPA3, RNF2, RFC4, RELB, RECK, RBBP6, RB1, RAD51, QSCN6, PTTG1, PTN, PTHLH, PRC1, PMP22, PLAGL1, PKD2, PGF, PDGFRA, PDGFB, PCNA, PCM1, PA2G4, ORC6L, NUSAP1, Nrp, NRAS, NME1, NFIB, NFIA, NEK2, NDN, MYC, MYB, MST1R, MKI67, MCM7, MCM5, MCM3, MAD2L1, LRP1, LIG1, KPNA2, KIT, KIF2C, KIF11, JUNB, ISG20, IL15, IGFBP6, IGF2, IGF1, IFRD2, IFNAR2, IFI16, Idb2, HK2, GTF2H1, GPS1, GPC4, Gnrh, GAS6, GAS1, FYN, FOS, FLT3, FLT1, FIGF, FGR, FEN1, F2R, ETS2, ERBB3, EPS15, ENPEP, EMP2, ELF5, EGFR, ECT2, DUSP6, DOCK2, DDIT3, DAB2, CYR61, CXCL1, CSF1R, CRIP2, CRIP1, CORO1A, COL18A1, CKS2, Cks1, CHEK1, CHAF1B, CDKN2C, CDKN1C, CDC6, CDC34, Cdc2a, CDC25A, CDC20, CD68, CCNG2, CCND2, CCND1, CCNB2, Ccnb1-rs1, CCNA2, Calml4, CALM3, BUB1, BTG3, BTG2, BTG1, BIRC5, BIN1, BAT3, AREG, APRIN, ANXA1, AK1, AIF1, AHR
Cell adhesion (82)	ADAM12, AEBP1, ALCAM, AOC3, APP, ARHC, BYSL, CCL2, CD34, CD36, CD44, CD47, CD9, CDH13, CDH2,CDH3, CDH5,ACAM1, Ceacam2,CELSR2,CNTN1,COL14A1,COL15A1,COL18A1,COL1A1, COL1A2,COL3A1,COL4A1,COL4A2 ,COL5A1,COL5A2,COL6A1,COL6A2,COL6A3,COL7A1,COL8A1,CSPG2,CTGF,CYR61,DDR2,DPT,DSC2,ICAM1,CAM2,ISLR,ITGA6,ITGA7,ITGB2, LAMA2,LAMA4,Lamb1-1,LAMC1,LAMC2,LGALS3,LGALS8,LGALS9,MPDZ,MRC1, NID2,Nrp,PCDH7,PKD2,PRLR,PTK7,PTPNS1,PTPRF,SGCE,SPP1,SRPX,STAT5A,STAT5B,TEK,TGFBI,THBS1,THBS2,TPBG,TSTA3,VCAM1,VWF
Angiogenesis (13)	COL18A1, TEK, Vegfa, THBS1, Agpt2, FIGF, CYR61, PGF, CTGF, SERPINE1, VEGFC, Nrp, FLT1
Blood vessel development	COL18A1,TEK,Vegfa,THBS1,Qk, Agpt2,FIGF,CYR61,PGF,CTGF,SERPINE1,VEGFC,Nrp,PPAP2B,FLT1
Cell cycle pathway	MCM3, MCM5, PTTG1, PCNA, TGFB3,CDC6, Trp53,CDKN2C,RB1, TGFB2,MCM7, CCNB2,CDKN1C,ORC6L,CDC20,CDC25A, CCND2,CCNA2,MAD2L1
Cell growth anddeath pathway	MCM3,MCM5,PTTG1,PCNA,CASP1,TGFB3,CDC6,Trp53,CDKN2C,BAD,RB1,TGFB2,MCM7,CCNB2,TNFRSF1B,CDKN1C, ORC6L,CDC20,CDC25A,CCND2,PTPN13,CCNA2,MAD2L1
VEGF receptor activity	PDGFRB, Nrp, PDGFRA, FLT3, FLT1, KIT

EASE score<0.05.

# genes with red word are cancer mutant gene identified in reference (Nat Rev Cancer,4(3):177).

### The 835 Prognosis Classifier Predicted Clinical Outcome in Three Groups of Breast Cancer Patients

**Figure 5:**
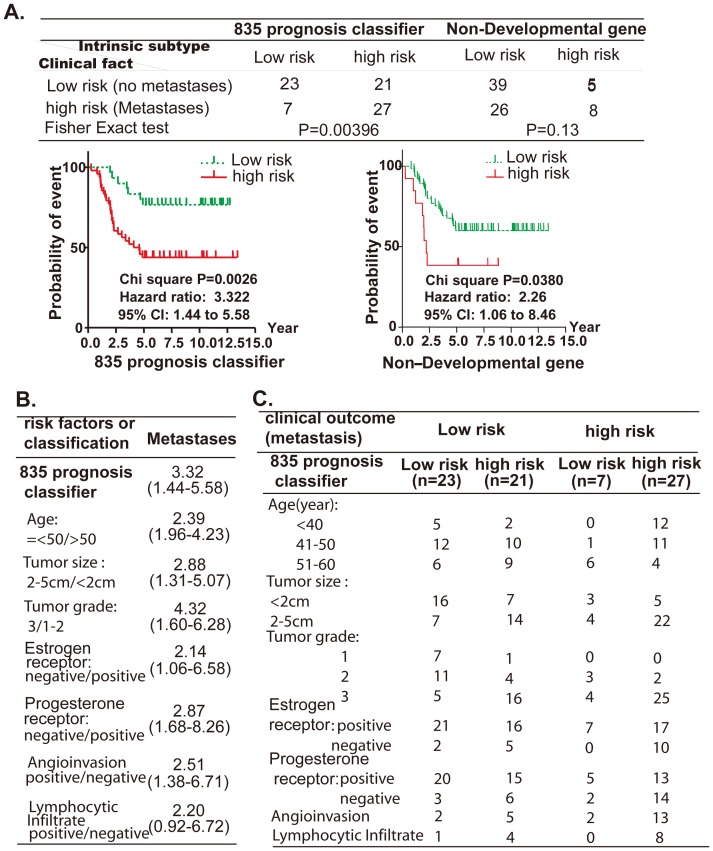
The 835 prognosis classifier acts as a powerful predictor of clinical outcome in 78 breast cancer patients. **A.** 78 breast cancer samples were first classified by the expression profiles of the 835 prognosis classifier, using unsupervised classification. The survival curve of the two groups was then compared with Kaplan Meier analysis to define clinical outcome (lower panel). Accuracy of classification was assessed with Fisher exact test (upper table). **B.** The distribution of tumors risk factors in the four groups classified by clinical metastasis and by the 835 prognosis classifier. **C.** The prognostic value of the 835 prognosis classifier and tumor risk factors.

Van’t Veer et. al. profiled gene expression in 78 breast cancer samples and defined an optimal prognosis classifier [8]. Following his protocol, the outcome of these 78 breast tumors were here predicted with the 835 prognosis classifier. First, all tumors were classed into two types by unsupervised classification with a hierarchical clustering algorithm. The survival curves of the two groups were then compared with Kaplan Meier analysis. The two groups showed clear difference in the probability of distant metastasis (hazard ratio 3.32, *p* value = 0.0026, Fig. 5A, left). Thus, the two groups were referred to as “low risk” and “high risk” groups. In total, 23 non-metastatic and 27 metastatic tumors were correctly predicted (50, 64.1% of the total 78 patients) (Fisher exactly test *P*<0.01) (Fig. 5A, left). In contrast, the mammary gland non-developmental-associated gene subset had a very poor ability to predict disease outcome (*P* = 0.13) (Fig. 5A, right).

Beside of metastasis, the clinical prognosis was also diagnosed with several pathologic characteristics, such as tumor size, histological grade, estrogen receptor expression (ESR1), etc. These pathologic factors can also predict the clinical outcome. Here, we also predicted the outcome of 78 patients with pathologic factors based on metastasis status, and then evaluated the distant metastasis hazard ratio between high and low risk patients classified by them or the 835 prognosis classifier. The results showed that the 835 prognosis classifier generated a high metastasis hazard ratio (3.3 compared with 2.0–2.8) (Fig. 5B). The 835 prognosis classifier, therefore, was more efficient at predicting clinical outcome in breast cancer patients than other clinical pathologic factors.

In the Van’t Veer study, clinical outcome of total 78 patients was simply classed as low or high risk based on tumors without or with metastasis. However, all tumors with low clinical outcome, still contained high percentage of tumors exhibited high risk pathologic characteristics, such as 2–5 cm size (21, 47.7% of total 44), histological grade 3 (21, 47.7% of total 44), negative ESR1 (7, 15.9% of 44). So, the clinical outcome prediction need more exact definition. Here, we refined the clinical outcome of 78 patients with our molecular prognosis classifier. Our prognosis classifier predicted total 21 tumors as high risk tumors form 43 low clinical risk tumors, and cleared out most of tumors with high risk pathologic characteristics, such as 2–5 cm size (14, 66.7% of total 21), histological grade 3 (16, 76% of total 21), negative ESR1 (5, 71.5% of 7), angioinvasion (5, 71.5% of 7), and lymphocytic infiltrate (4, 80% of 5) (Fig. 5C). As a result, the high risk group defined by the prognosis classifier (48, 61.5% of total 78) is more exactly and covered most of tumors with high risk characteristics, such as distant metastasis (27, 79.4% of total 34), 2–5 cm size (36, 76.6% of total 47), histological grade 3 (41, 82% of total 50), negative ESR1 (15, 88.2% of 17), angioinvasion (18, 81.8% of 22), and lymphocytic infiltrate (12, 92.3% of 13) (Fig. 5C). Thus, our prognosis classifier showed high efficiently to classify the high risk tumor from the low clinical outcome tumors class.

**Figure 6:**
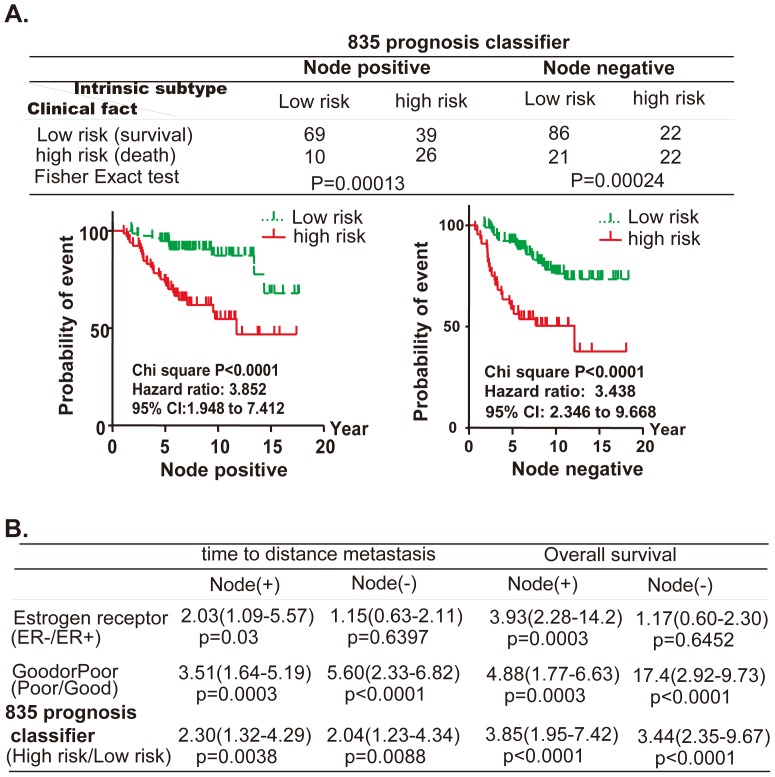
The 835 prognosis classifier could predict clinical outcome in a large set of breast cancer patients. The intrinsic dataset was applied to 144 node positive and 151 node negative primary breast tumors. The accuracy of prediction (**A**) or the prognosis value (**B**) of 835 prognosis classifier and tumor risk factors was assessed by the same approach as described in the legend of Fig. 5B.

To further validate the efficiency of outcome prediction by the 835 prognosis classifier, we pooled 144 node-positive and 151 node-negative breast tumors [7]. As expected, the 835 prognosis classifier successfully classified the tumors into two groups with a clearly different probability of survival (Node-positive: hazard ratio 3.85, *p* value<0.0001; Node-negative: hazard ratio 3.44, *p* value<0.0001, Fig. 6A). The percentage of correct prediction of clinical outcome approached 70% (Node-positive: 95, 66% of total 144, *p* value = 0.00013; Node-negative: 108, 72% of 151, *p* value = 0.00024, Fig. 6A). We also assessed the hazard ratio for distant metastasis or overall survival in high versus low risk patients, as defined by the 835 prognosis classifier, compared to tumor risk factors. The prognostic value of the 835 prognosis classifier was stronger than that of the estrogen receptor and had a higher hazard ratio for distant metastasis and overall survival, especially for patients with node negative tumors (2.04 ∝1.15, 3.44 ∝1.17, respectively, Fig. 6B).

## Discussion

In this study, we systemically analyzed on a global scale the relationship between gene expression changes during mammary gland development and breast tumorigenesis, and found that the two processes shared gene expression patterns. This suggested that, to a certain extent, similar molecular mechanisms may be used during both processes. Based on commonality in gene expression, we successfully defined an “835 prognosis classifier” subset as a prognostic classifier that is more powerful at prognosis prediction than most tumor risk factors (e.g. histological grade, ESR1) in current use (Fig. 5B, Fig. 6B).

The fact that organ development and neoplasia share gene regulatory mechanisms has been known for a long time [18], [19]. However, most previous studies explored the expression of single genes or a small group of genes, thus not revealing the global picture [21], [22]. Recently, several studies found that gene expression changes during normal tissue development are frequently recapitulated in during neoplasitc transformation of the same tissue using global computation. This includes studies in the lung [37], [38], the intestine [24], the liver [39], and the cerebellum [23]. Our present study discovered that in the mammary gland, a unique organ that exhibits a linear phase of development followed by cyclical phases, there are a shared molecular mechanisms between organ development and tumor formation. The early diagnosis and prediction of prognosis are important steps for the effective treatment of cancer, not the least in breast cancer [40]. In the past twenty years, the death rate for breast cancer patients has dropped as improvements occur in early diagnosis and outcome prediction [40]. However, these advances have not gone far enough to eliminate the morbidity and mortality of breast cancer. Classically, histological information is a major criterion for the diagnosis and prognosis in breast cancer [40], [41]. Unfortunately, histological classification cannot support optimal clinical decisions due to several shortcomings [40], [41]. For example, histological grade 2 contains both low and high risk patients [11]. In addition, the typical histological character is not always obvious and there is an element of subjectivity involved [37]. Therefore, new approaches will benefit on the improvement for the clinical classification of breast cancer.

Breast tumorigenesis is thought to be a step-wise mutation process that leads to changes in gene regulation. Consequently, changes in gene expression pattern are associated with the tumor developmental process, and these changes can potentially be used to classify the tumor type and stage. A numbers of research groups are therefore now trying to derive small gene subsets from genome-wide profiling studies on cancer tissues to improve the early diagnosis and prognosis prediction [7], [8], [11], [31], [33], [42]–[46]. These studies have already defined several prognostic sets that have proved promising for prognosis prediction [3], [4], [11]. Previous studies [23], [24] and the current study demonstrate that organ development exhibits partially overlapping gene expression patterns with tumorigenesis. Therefore, organ developmental genes can also be used to refine clinical prognosis classifiers. Recently, a study has successfully predicted the survival of human lung cancer based on gene expression patterns associated with mouse lung development [37]. By combining gene expression profiles characteristic for normal mouse mammary gland development and for breast cancer, we were able to define a smaller subset of genes that show a good value to efficiently predict the breast cancer clinic prognosis. Interestingly, this classifier geneset contains at least 30 common genes with the 231 outcome associated genes and 12 common genes with 70-genes optimal signature identified by van’t Veer et al [8]. Sotiriou et al reported a signature with 183 genes, 97 of which are specifically associated with tumor grade (1&3) [11]. We found that 60 of these 183 genes were also in our list. Thus, our study contributes additional evidence in support of this concept by demonstrating that mammary gland developmental genes are useful for the prediction of breast cancer prognosis.

Although many cancer-causing genes have been identified, the high degree of tumor heterogeneity suggests that additional genes and mechanisms remain to be discovered [3], [4], [6], [17]. The identification of these additional genes will be important because cancer therapy is likely to be increasingly tailored to molecular abnormalities in tumors of individual patients [6]. Organ development and tumor formation share several cellular processes, and consistent with this idea, an overlap in gene regulation between the two processes is suggested by our study in the mammary gland and by previous work in other organs [23], [24], [38]. These studies also suggest that developmental genes are a potential pool of new cancer genes and that mechanisms can be identified by globally analyzing the co-expression of genes across cerebellar, lung, intestine, and liver development, and the cancers derived from these organs [23], [24], [38]. The mammary gland is a unique developmental organ. Its developmental progress contains not only a linear phase in the embryonic and early postnatal period, but also has a cyclical phase governed by many hormones, growth factors, and transcriptional proteins in the adult [30]. Perturbation of these regulatory genes may lead to mammary gland neoplasia. Our prognosis genes set was enriched in genes involved in carcinogenesis, such as Trp53, Myc, Kit, RB1, EGFR, CCND1, and CCND2 [36], as well as cancer related growth factor signaling molecules such as TGFβ, IGF1, PDGF, EGF and VEGF. This approach, therefore, may be a suitable source to identify new breast cancer genes.

This study provides the first global insights into the molecular mechanisms that are conserved between mammary gland development and breast cancer. Based on these insights, a novel multiscale analysis model has been derived to better predict the prognosis outcome in human breast cancer. These results also suggest that developmental roles of genes may be important criteria for selecting genes for prognosis prediction in breast cancer. Of course, our prognosis genes set is not perfect and has some flaw to be improved in the future work. Such as, our prognosis genes set have gotten from gene transcriptional change and didn’t cover the information of post-translational modifications of signaling proteins. But post-translational modifications were important in both mammary gland development and breast tumorigenesis. Thus, the combination with epigenetic information will improve the prognosis of breast cancer.

## Materials and Methods

### Selection of Mouse Mammary Gland Gene Expression Datasets

To identify datasets in which expression profiling is associated with mammary gland developmental cycles, we queried PubMed (http://www.ncbi.nlm.nih.gov/sites/entrez) and found several gene expression microarray databases for mouse mammary glands [31], [47]–[49]. The database of Rodolph et al 2003 [31] was associated with mammary gland cycles (File S8) and contains gene expression profiles for virgin (Vir), for pregnancy at day1 (P1), day3 (P3), day7 (P7), days12 (P12) and days17 (P17), for lactation at day1 (L1), day2 (L2), day9 (L9), and for a forced weaning point at day 2 (invol2) [31].

### Collection of Gene Expression Databases for Human Primary Breast Cancer

To capture comprehensive expression datasets of primary breast tumor, we queried all studies on well-characterized breast cancer from web sites Oncomine (http://141.214.6.50/oncomine) and GEO datasets (http://www.ncbi.nlm.nih.gov/gds). This search yielded four adequate microarray databases containing “altered” genes, meaning genes that have significantly high or low expression in primary breast cancer [7], [8], [15], [33] compared to that in the reference sample pool. In addition, we collected three other mammary gland tumor gene profiling databases to evaluate the prediction of clinical outcome [11], [34], [35], Basic on the gene profiling in these primary database, several small number of prognostic prediction gene sets have been developed in previou research, such as MammaPrint [8], Mapquant [11], and Theros.

For the present study, we only considered human genes that had a mouse homologue, as specified through the HomoloGene database. The human probe ID and Locus ID of each mouse mammary gland associated gene was also identified.

### Collection of the Literature-based Cancer Modulated Genes

A literature-based cancer modulated gene list has been generated based on three websites (www.infobiogen.fr/services/chromcancer/index.htm, www.cancerindex.org/geneweb and condor.bcm.tmc.edu/oncogene.html) where genes have been shown to mediate cancer initiation/progression, as supported by at least one published evidence. File S4 lists a literature-based cancer modulated genes that have mouse probe ID in the Mu72AV2 chip.

### Calculation of Odds Ratio

The odds ratio is the ratio of the odds of an event occurring in one group to the odds of it occurring in another group. Here, we calculated the odds ratio of literature-based mammary gland-cycle associated genes in the developmentally and non-developmentally associated genes subset. If the probabilities of literature-based mammary gland-cycle associated genes in the developmentally and non-developmentally associated genes subset. are *p*_1_ and *p*_2_, respectively, then the odds ratio is: (p1/(1-P1))/(p2(1-P2)).

### Principal Component Analysis (PCA) of Developmentally Associated Genes

Before we performed temporal PCA of the mouse data, each of the 2939 genes was individually normalized to mean zero and variance one across all time points [50]. PCA was performed using Matlab. The percentage of temporal variances captured by each of the first five temporal PCs (of 18 nonzero components) was 49.28%, 26.98%, 8.90%, 3.92%, and 3.15%, totaling 92.22%.

### Enrichment Assay

Overrepresented gene categories were identified by EASE software (Expression Analysis Systematic Explorer, available at http://david.niaid.nih.gov/david/ease.htm), EASE score <0.05. The experiment was performed according to the protocol of the EASE software.

### Classification of Tumor to Predict Clinical Outcome

The experiment perform following the method and protocol in reference (Van’t Veer et. al., 2002) [8]. All tumors were classed into two groups with genes classifier genesets by unsupervised classification with a hierarchical clustering algorithm. Then, the clinical outcome of tumor in each class was analyzed based on metastasis status. Generally, the class contained more metastasis’tumor was thought as a “poor progonosis” tumor class, while another class included more no-metastasis’s tumor was be regard as a “good progonosis” class. At last, the predict efficiency was assessed based on the percentage of total tumor which actual outcome was correctly predicted.

## Supporting Information

File S1 **Literature-based mammary gland-cycle associated genes.** All gene expression was demonstrated to associate with one or more stages of the mammary gland development at the mRNA or protein level in published research.
(XLS)

File S2 **Presented genes and developmentally associated genes.** These genes were filtered and refined systematically with different cutoffs from a mouse mammary gland epithelial gene expression profiling database, following the process shown in Fig. 1A.The details are described in the text.
(XLS)

File S3 **Enrichment of ontology in developmentally associated genes.** Overrepresented gene categories were identified by EASE software (Expression Analysis Systematic Explorer, available at http://david.niaid.nih.gov/david/ease.htm), EASE score <0.05.
(XLS)

File S4 **Literature-based cancer modulated genes.** The literature-based cancer modulated genes are genes that mediate cancer progress, as supported by at least one published piece of evidence. This list is based on three websites: (www.infobiogen.fr/services/chromcancer/index.htm, www.cancerindex.org/geneweb, and condor.bcm.tmc.edu/oncogene.html). Cancer genes that have mouse probe IDs in the Mu72AV2 chip are listed.
(XLS)

File S5 **The Principal Component assay on the developmentally associated genes.** The percentage temporal variances captured by each of the first five temporal PCs (of 18 non zero components) were 49.28, 26.98, 8.90, 3.92, and 3.15%, totaling 92.22%.
(XLS)

File S6 **The 835 prognosis classifier.** Genes in this list were altered in expression in at least two primary breast cancer databases. The details are described in the text.
(XLS)

File S7 **Enrichment of ontology in the 835 prognosis classifier.** Only processes and pathways in which EASE score was less than 0.05 are listed here.
(XLS)

File S8 **The expression pattern of milk genes in three databases of gene expression profiling in mouse mammary gland.** Seven milk genes were selected based on the published references. Three mouse mammary gland microarray databases were collected from Pubmed website. The curve line in each figure shows the relative expression pattern of each gene in mammary gland development.
(TIF)
