# Supplementary figures and images for: A Prognosis Classifier for Breast Cancer Based on Conserved Gene Regulation between Mammary Gland Development and Tumorigenesis: A Multiscale Statistical Model
Source: PLoS One. 2013 Apr 2;8(4):e60131. doi: 10.1371/journal.pone.0060131 (PMC3614930; doi:10.1371/journal.pone.0060131)

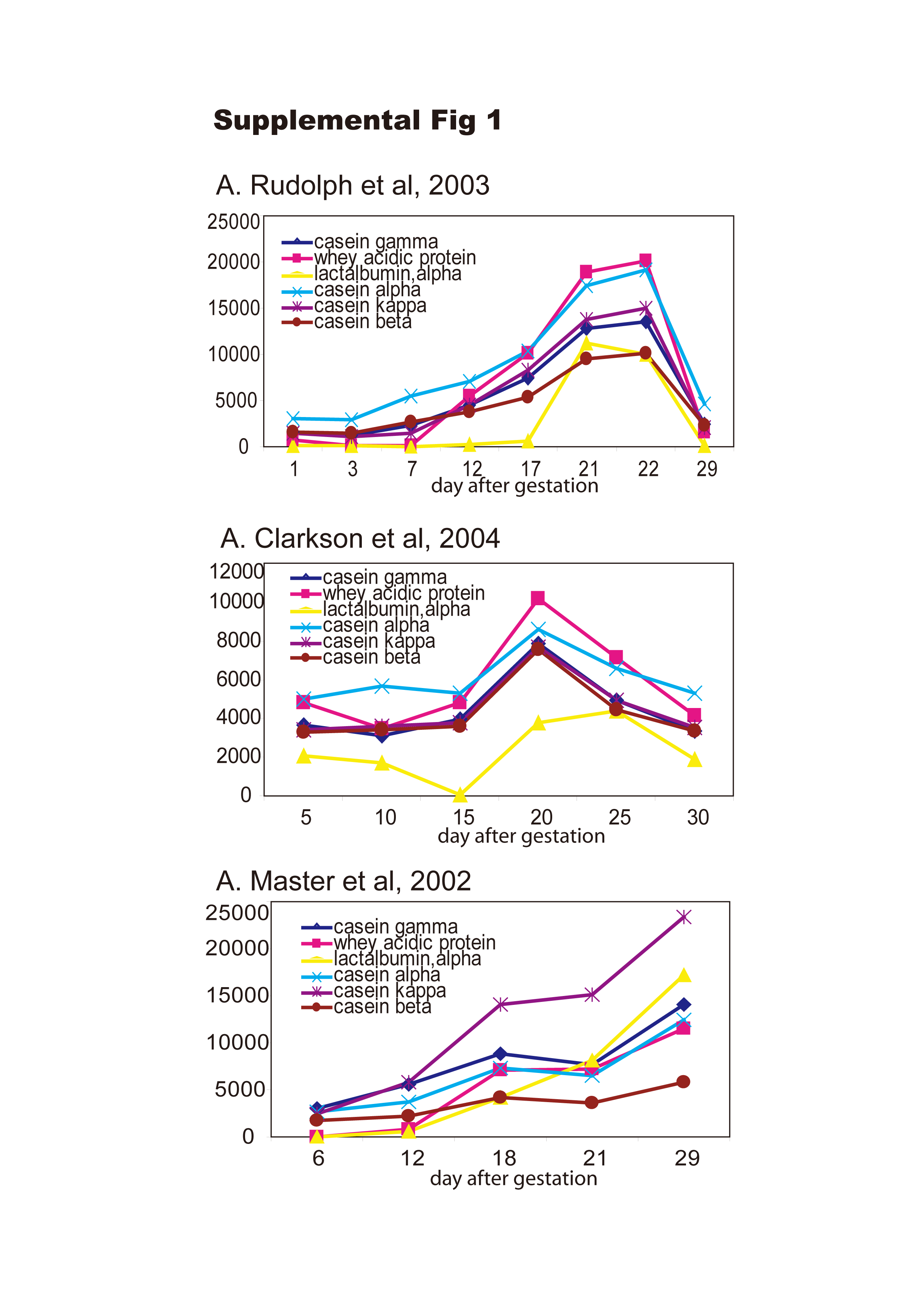

Supplement: File S8 — The expression pattern of milk genes in three databases of gene expression profiling in mouse mammary gland. Seven milk genes were selected based on the published references. Three mouse mammary gland microarray databases were collected from Pubmed website. The curve line in each figure shows the relative expression pattern of each gene in mammary gland development. (TIF) [file pone.0060131.s008.tif]
